# The DEAD-box Protein Rok1 Orchestrates 40S and 60S Ribosome Assembly by Promoting the Release of Rrp5 from Pre-40S Ribosomes to Allow for 60S Maturation

**DOI:** 10.1371/journal.pbio.1002480

**Published:** 2016-06-09

**Authors:** Sohail Khoshnevis, Isabel Askenasy, Matthew C. Johnson, Maria D. Dattolo, Crystal L. Young-Erdos, M. Elizabeth Stroupe, Katrin Karbstein

**Affiliations:** 1 Department of Cancer Biology, The Scripps Research Institute, Jupiter, Florida, United States of America; 2 Department of Biological Science and the Institute of Molecular Biophysics, Florida State University, Tallahassee, Florida, United States of America; 3 The Benjamin School, Palm Beach Gardens, Florida, United States of America; University of California, Berkeley, UNITED STATES

## Abstract

DEAD-box proteins are ubiquitous regulators of RNA biology. While commonly dubbed “helicases,” their activities also include duplex annealing, adenosine triphosphate (ATP)-dependent RNA binding, and RNA-protein complex remodeling. Rok1, an essential DEAD-box protein, and its cofactor Rrp5 are required for ribosome assembly. Here, we use in vivo and in vitro biochemical analyses to demonstrate that ATP-bound Rok1, but not adenosine diphosphate (ADP)-bound Rok1, stabilizes Rrp5 binding to 40S ribosomes. Interconversion between these two forms by ATP hydrolysis is required for release of Rrp5 from pre-40S ribosomes in vivo, thereby allowing Rrp5 to carry out its role in 60S subunit assembly. Furthermore, our data also strongly suggest that the previously described accumulation of snR30 upon Rok1 inactivation arises because Rrp5 release is blocked and implicate a previously undescribed interaction between Rrp5 and the DEAD-box protein Has1 in mediating snR30 accumulation when Rrp5 release from pre-40S subunits is blocked.

## Abstract 2

During ribosomal biogenesis, Rrp5 is unusual in being required for assembly of both small and large subunits. This study demonstrates a role for ATP hydrolysis by the DEAD-box protein Rok1 in releasing Rrp5 from pre-40S subunits.

## Author Summary

Assembly of the small and large ribosomal subunits requires two separate machineries. The assembly factor Rrp5 is unusual in being one of only three proteins required for assembly of both subunits. While it binds cotranscriptionally during early stages of small subunit assembly, it departs with large subunit intermediates after the separation of these precursors. How Rrp5 switches from interacting with small subunit precursors to binding large subunit precursors remains unknown but is potentially important, as it could regulate the interplay between small and large subunit assembly. Here, we show that the DEAD-box protein Rok1, a member of a ubiquitous class of RNA-dependent ATPases, releases Rrp5 from assembling small subunits to allow for its function in large subunit assembly. We show that a complex of Rrp5, Rok1, and adenosine triphosphate (ATP) binds small subunits or mimics of ribosomal RNA more tightly than does a complex of Rrp5, Rok1, and adenosine diphosphate (ADP). In cells, interconversion between the ATP and the ADP-form of Rok1 is required for release of Rrp5 from nascent small subunits and for binding to assembling large subunits. Furthermore, we show that the release of snR30, which leads to formation of a large substructure on small subunits, also requires Rok1-mediated release of Rrp5.

## Introduction

Ribosome assembly involves the transcription, modification, and processing of a large precursor rRNA, encoding 18S, 5.8S, and 25S rRNAs separated by spacer regions (internal transcribed spacers [ITSs]) (S1A and S1B Fig). These processes are integrated with the binding of ribosomal proteins and rRNA folding via the activities of a large machinery comprising ~200 assembly factors (AFs), most of which are essential [1]. However, because pre-40S subunits are nucleolytically separated from pre-60S subunits before the latter assembles [2,3], two completely different sets of AFs are required for 40S and 60S ribosome assembly.

Rrp5, a 193 kDa protein, is one of three AFs that function in the biogenesis of both subunits. It consists of 12 tandem repeats of the S1 domain, followed by seven tetratricopeptide (TPR) repeats (S1C Fig). The S1 domain, first found in the ribosomal protein S1, is an RNA-binding motif from the oligonucleotide binding fold (OB-fold) family. Like many RNA-binding motifs, it appears that OB-folds can recognize ligands other than RNA [4–7]. TPR motifs are known as protein-protein interaction sites [8].

Binding relatively early during rRNA processing, Rrp5 is required for the recruitment of a subset of AFs required for 40S ribosome assembly, including the DEAD-box protein Rok1 [9,10]. After its function in 40S subunit assembly is complete, Rrp5 remains bound to pre-60S subunits, thus explaining its requirement for 60S assembly [7,11]. This highly unusual observation—binding to pre-40S ribosomes yet departing with pre-60S ribosomes—was apparently explained by Rrp5 binding to ITS1, the spacer region between 18S and 5.8S rRNA (S1B Fig). Specifically, quantitative RNA binding experiments demonstrated that interactions with the regions of ITS1 that ultimately become part of pre-60S subunits are much stronger than its interactions with ITS1 regions that become part of pre-40S subunits [12]. This explains Rrp5 binding to pre-60S subunits after these ITS1 regions are separated by endonucleolytic cleavage during assembly. Nevertheless, recent cross-linking experiments also suggest the existence of a binding site for Rrp5 within 40S subunits [13]. This finding, if confirmed, would indicate that departure of Rrp5 from pre-40S subunits requires dissociation of Rrp5•40S interactions. How such dissociation is promoted remains unclear.

Rrp5 interacts directly with the DEAD-box protein Rok1 and is required for its RNA specificity [14]. DEAD-box proteins have often been referred to as RNA helicases; however, their functions also include duplex annealing, adenosine triphosphate (ATP)-dependent RNA binding, and protein displacement [15–18]. Indeed, recent enzymatic analyses suggest that DEAD-box proteins have limited processivity as helicases [19,20]. Rok1 has been previously implicated in 40S ribosome assembly [21]. Its depletion affects the earliest steps of 40S ribosome assembly and leads to snR30 accumulation in pre-40S intermediates [22]. However, snR30, an essential small nucleolar RNA (snoRNA), also accumulates on pre-40S upon depletion of the DEAD-box protein Has1 [23] and the AF Utp23 [24]. Interestingly, in vitro experiments do not show strong helicase activity for Rok1 [14,25] but demonstrate duplex annealing activity [14].

Here, we use a combination of biochemical experiments and yeast analyses to show that *Saccharomyces cerevisiae* Rrp5 interacts with the 40S subunit. This interaction is strengthened by Rok1 in the ATP-bound form, but not the adenosine diphosphate (ADP)-bound form, suggesting that interconversion by ATP hydrolysis leads to release of Rrp5 from pre-40S ribosomes. Indeed, we show that ATP hydrolysis by Rok1 is required for Rrp5 release from pre-40S subunits and departure with pre-60S subunits in vivo. Structural studies by single-particle three-dimensional electron microscopy (3DEM) and X-ray crystallography, combined with biochemical experiments, demonstrate that allosteric communication between Rok1 and 40S subunit binding involves the central S1 domains of Rrp5. Finally, we demonstrate that the accumulation of snR30 in pre-40S subunits upon Rok1 inactivation arises because Rrp5 release is blocked and appears to be mediated by the simultaneous accumulation of the DEAD-box protein Has1.

## Results

### Rrp5 Binds 40S Subunits

Recent in vivo cross-linking studies confirmed previous quantitative RNA binding studies from our lab suggesting that ITS1 is a major Rrp5 binding site [12,13]. Surprisingly, however, this study also uncovered a cross-link for Rrp5 within H27 of 18S rRNA [13]. This is consistent with recent findings that Rrp5•Rok1 is found in assembling 40S subunits once the central domain, which contains H27, is transcribed [26]. These data, if confirmed, would suggest that after separation of pre-40S and pre-60S rRNAs by Rcl1-dependent cleavage, Rrp5 would need to be actively dissociated from pre-40S subunits in order to depart with pre-60S ribosomes as previously observed [7,11]. Because the cross-link in 18S rRNA was relatively weak, we sought to verify the interaction between Rrp5 and 40S subunits using recombinant Rrp5 and mature 40S subunits.

**Fig 1:**
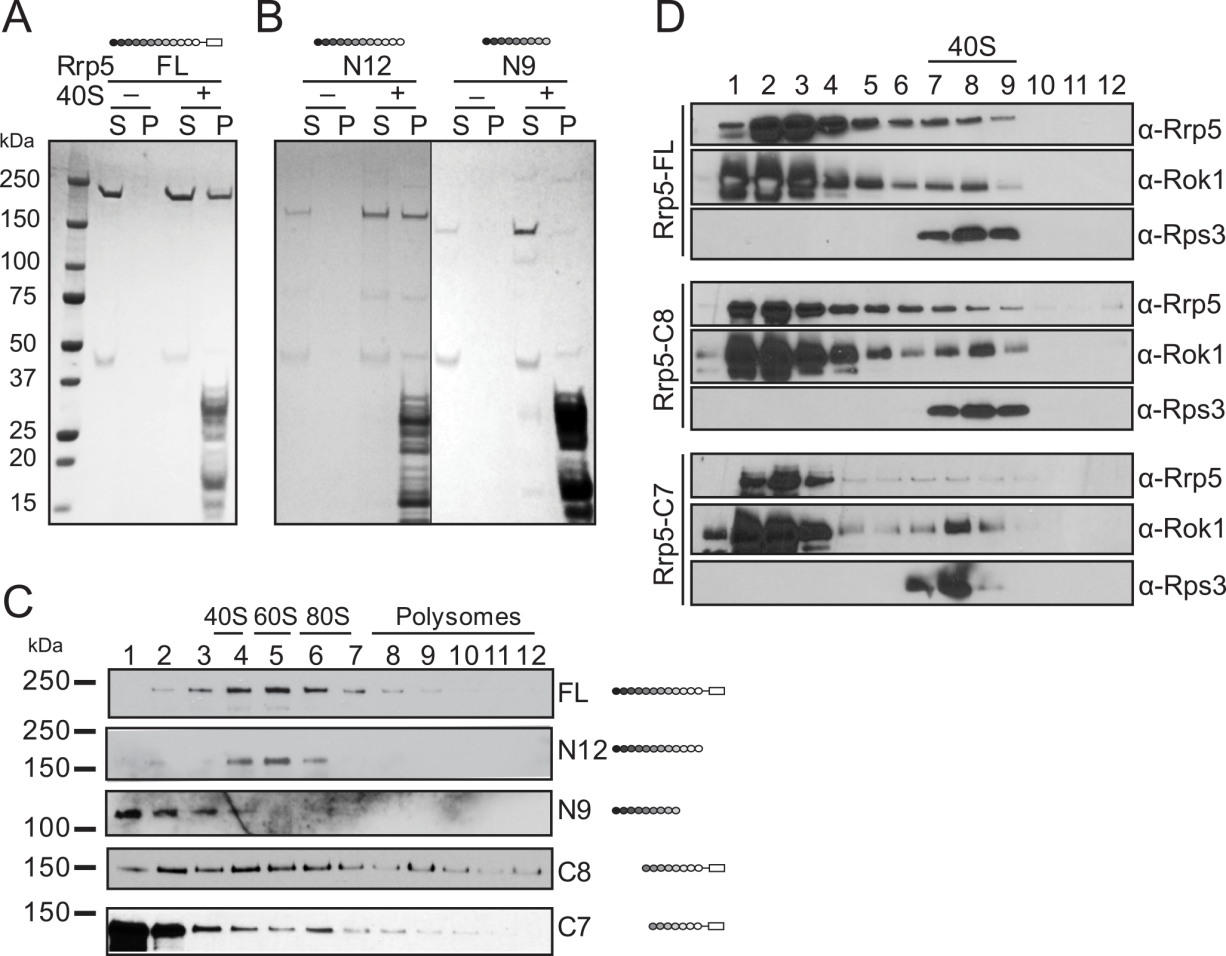
Rrp5 binds to 40S ribosomal subunits in vivo and in vitro. (A) Ultracentrifugation pelleting experiments demonstrate that recombinant full-length Rrp5 binds purified mature 40S subunits in vitro. S, supernatant; P, pellet. (B) The three C-terminal S1 domains are essential for the interaction between Rrp5 and 40S subunits in vitro. Recombinant Rrp5 fragments are used in the same pelleting experiment as 1A. (C) Gradient centrifugation demonstrates a role for the three C-terminal S1 domains and S1–S5 for binding to preribosomes in vivo. (D) Gradient sedimentation experiments of various recombinant Rrp5•Rok1•AMPPCP complexes demonstrate that S1–S5 contributes to Rrp5 binding to 40S ribosomes in vitro. Note that the more sensitive gradient sedimentation experiments are required to demonstrate the more quantitative than qualitative differences in Rrp8_C8 and Rrp5_C7 binding. Because Rrp5_N9 and Rrp5_N12 do not bind Rok1, binding of these fragments requires the pelleting assay. All experiments were repeated at least twice, and representative data are shown.

We incubated Rrp5 with and without 40S ribosomes before sedimentation through a sucrose cushion (Fig 1A), followed by analysis of the pellet and the supernatant for the presence of Rrp5 by sodium dodecyl sulfate polyacrylamide gel electrophoresis (SDS-PAGE). While Rrp5 does not pellet by itself, addition of 40S ribosomes leads to its recovery in the pellet, indicating that Rrp5 binds mature 40S subunits (Fig 1A). Because this pelleting experiment tends to overestimate binding affinities (as 40S-bound Rrp5 is constantly removed from the equilibrium through pelleting), we wanted to confirm this finding in a more sensitive and reliable gradient sedimentation assay. The complex was therefore sedimented through a 5%–20% glycerol gradient, which separates 40S bound proteins from free proteins, before analysis of the fractions by western blotting against Rrp5 and Rok1. The position of 40S subunits is marked by western blotting against Rps3 and the ultraviolet (UV) absorbance of 18S rRNA. Importantly, the data in Fig 1D show that in the presence of Rok1 and a nonhydrolyzable ATP analog, AMPPCP, Rrp5 binds and comigrates with the 40S ribosomal subunits.

We used 40S ribosomes in these experiments because we wanted to ascertain that Rrp5 could bind to the assembling 40S structure and exclude the possibility that interactions with assembly intermediates could reflect interactions with the known binding sites in ITS1. Furthermore, mature 40S subunits are more readily available in the quantities required for this experiment than early pre-40S precursors. Importantly, such reconstituted experiments are analogous to others reported for 60S AFs [27,28] or the bacterial homolog of the AF Dim1 [29]. Nevertheless, to validate that the interactions between mature 40S subunits and Rrp5 are similar to those between early pre-40S ribosomes and Rrp5, we determined whether they depended on the same domains in Rrp5 (see S1C Fig for a schematic representation of Rrp5).

Removal of the C-terminal TPR domains (Rrp5_N12, see S1C Fig for an explanation of the nomenclature used to describe the Rrp5 truncations) does not affect Rrp5 binding to 40S ribosomes in vitro or pre-40S ribosomes in vivo (Fig 1B and 1C). In contrast, removal of the TPR domains and the three C-terminal S1 domains (Rrp5_N9) weakens Rrp5 binding to 40S ribosomes in vitro (Fig 1B) and to pre-40S ribosomes in vivo (Fig 1C). These data indicate that the three C-terminal S1 domains are important for Rrp5’s interactions with pre-40S ribosomes.

Similarly, N-terminal truncation of the first four S1 domains of Rrp5 to produce Rrp5_C8 has little effect on Rrp5 binding to 40S ribosomes in vitro (Fig 1D) and allows for binding to preribosomes in vivo (Fig 1C). In contrast, removal of an additional S1 domain to produce Rrp5_C7 weakens Rrp5 binding to both mature 40S ribosomes in vitro (Fig 1D) and preribosomes in vivo (Fig 1C), such that now most Rrp5 remains unbound. These data suggest that the fifth S1 domain contributes contacts between Rrp5 and 40S subunits. Together, these data indicate that the interactions between Rrp5 and mature 40S ribosomes are specific and reflect on interactions between Rrp5 and early pre-40S ribosomes, thereby validating the use of mature 40S ribosomes for these biochemical studies. Furthermore, these data are also fully consistent with and further support in vivo cross-linking studies that uncovered cross-links between Rrp5 and 40S subunits [13], as well as in vivo assembly studies [26].

### Rok1•ATP but Not Rok1•ADP Binding Strengthens Rrp5 Binding to 40S Ribosomes

The finding that Rrp5 interacts with 40S ribosomes is surprising because Rrp5 remains bound to pre-60S after the initial rRNA cleavage that separates the precursors for the small and large subunits [7,11]. Furthermore, 40S assembly and 60S assembly proceed separately. To reconcile these observations, we hypothesized that Rrp5’s interactions with pre-40S subunits must be actively disrupted, otherwise Rrp5 would bridge these two now separate rRNAs and keep them together, at a time when pre-40S and pre-60S subunits are matured separately. DEAD-box proteins can dissociate short RNA duplexes, as well as remodel RNA-protein complexes [15,16]. We thus tested if Rok1 could be involved in removal of Rrp5 from pre-40S ribosomes, to allow for departure with pre-60S ribosomes.

**Fig 2:**
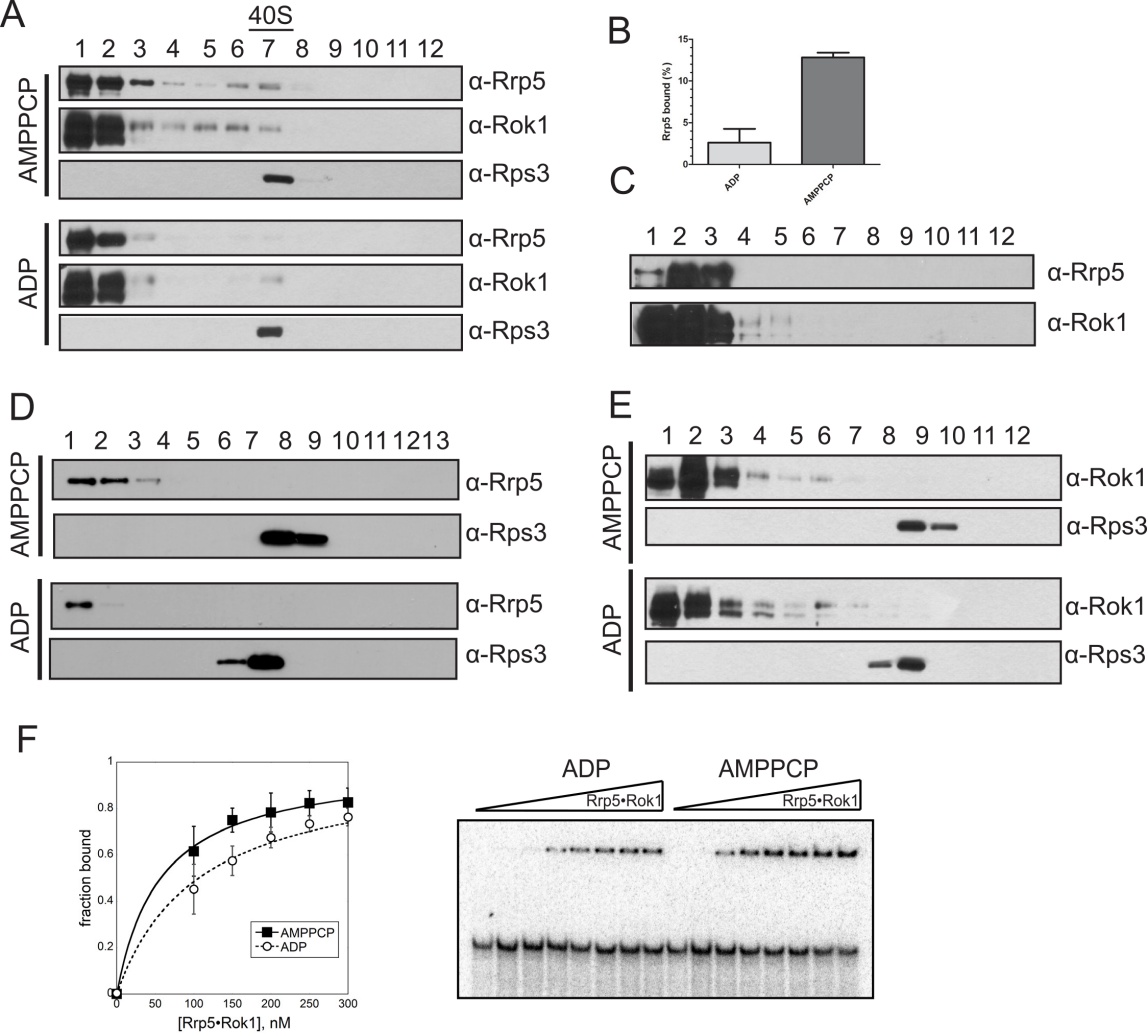
Rok1•Rrp5 binds to 40S subunits in the ATP-bound form. (A) Glycerol density gradient followed by protein precipitation and western blotting was used to analyze the binding of recombinant Rrp5•Rok1 to 40S subunits in the presence of AMPPCP or ADP. Fractions 1 and 13 represent the top (5% glycerol) and the bottom (20% glycerol) of the gradient, respectively. (B) Quantitation of the data in panel A and S2B Fig. The data are averages from two biological replicates. The numerical data underlying this graph can be found in S1 Data. (C) In the absence of 40S ribosomes, neither Rok1 nor Rrp5 enter the gradient, demonstrating that cosedimentation with 40S ribosomes reflects 40S binding. (D) Control gradients in the absence of Rok1 demonstrate that the AMPPCP- and ADP-dependent effects are mediated by Rok1. (E) Rok1 alone does not bind to 40S subunits in the presence of either AMPPCP or ADP. All experiments were repeated at least twice, and representative gels are shown. (F) Electrophoretic mobility shift assay of Rrp5_C7•Rok1 to H44-A_2_ rRNA mimics in the presence of AMPPCP or ADP. The left panel shows quantitation of data on the right, fit with a single binding isotherm to yield *K*_1/2_ values for the AMPPCP and ADP states of 57 ± 7 nM and 106 ± 24 nM, respectively. The numerical data underlying this graph can be found in S1 Data. Error bars come from three independent experiments.

To test if there could be a role for Rok1 in Rrp5 release, we developed an in vitro assay for Rrp5 release from 40S subunits. We assembled Rrp5•Rok1 complexes, mixed them with 40S ribosomes in the presence of ADP or the nonhydrolyzable ATP analog AMPPCP, and assayed 40S binding in a gradient sedimentation experiment. Importantly, control experiments demonstrate that Rrp5•Rok1 form a complex regardless of the nucleotide state (S2A Fig). The data show that in the AMPPCP-bound form, ~15% of Rrp5 is bound to 40S subunits (Fig 2A and 2B and S2B Fig). In contrast, in the ADP-bound form, Rrp5 sediments faster than 40S subunits, indicating that 98% of Rrp5 is free (Fig 2A and 2B and S2B Fig). Control experiments show that in the absence of 40S subunits, Rrp5 and Rok1 sediment on top of the gradient, demonstrating that the portion that enters the gradient is 40S subunit bound (Fig 2C). Additional control experiments indicate that nucleotide-dependent Rrp5 binding to 40S ribosomes depends on Rok1 because Rrp5 binding alone is not affected by addition of nucleotide (Fig 2D). These experiments also reveal that Rrp5 alone does not bind strongly enough to 40S subunits to survive the gradient sedimentation experiment, indicating that binding of Rok1 stabilizes Rrp5’s interaction with 40S subunits.

Note that in the pelleting experiments of Fig 1A, no Rok1 is added, therefore seemingly showing a different result. This variation arises from the difference between the assays. In the pelleting experiment of Fig 1A, 40S-bound Rrp5 is constantly removed from the equilibrium, thereby leading to overestimates of the binding. It was necessary to carry out the experiment of Fig 1B using this pelleting experiment, as TPR-less Rrp5 does not bind Rok1 and therefore does not bind 40S subunits in the gradient sedimentation experiment. Importantly, the overestimation of binding interactions in the pelleting experiment could also mask small effects arising from deletion of the TPR motif.

Finally, in the presence of Rrp5, Rok1 was also bound to 40S ribosomes in the AMPPCP-bound form, but not the ADP-bound form (Fig 2A). Nevertheless, Rok1 alone was unable to bind 40S subunits in either the AMPPCP- or the ADP-bound form (Fig 2E).

Together, these experiments indicate that Rrp5 and AMPPCP•Rok1 bind cooperatively to 40S ribosomes, while this cooperativity is lost with ADP-bound Rok1. Because AMPPCP is an ATP analog, these findings implicate a role for ATP hydrolysis, which interconverts these forms, in release of Rok1 and Rrp5 from pre-40S subunits.

### ADP Weakens the Interaction of Rrp5•Rok1 with Pre-18S rRNA

To further confirm the observation that the ADP-bound form of Rok1 releases Rrp5 from pre-40S ribosomes, we reconstituted an in vitro system to quantify the effects of ADP and AMPPCP binding on binding of the Rok1•Rrp5 complex to pre-rRNA mimics. In these experiments, increasing concentrations of Rrp5•Rok1 complex were mixed with a pre-18S rRNA mimic encompassing H44 to A_2_ (H44-A_2_, see S1B Fig) in the presence of either ADP or AMPPCP. Free RNA was separated from protein-bound RNA by native gel electrophoresis.

We were unable to use full-length Rrp5 in this experiment, as it binds more tightly to pre-rRNA mimics than to Rok1 [12,14]. Thus, under the subsaturating conditions required to reliably measure the RNA binding equilibrium, full-length Rrp5 and Rok1 do not form a complex. We therefore took advantage of the observation that Rrp5_C7 binds Rok1 well (see below) but displays weaker RNA binding (S2C Fig). Thus, under the conditions shown here, most Rrp5 and Rok1 form a complex. Our finding suggests that ADP weakens the binding of Rrp5_C7•Rok1 for H44-A_2_ by nearly 2-fold (*K*_1/2_ = 57 ± 7 nM for AMPPCP versus *K*_1/2_ = 106 ± 24 nM for ADP, Fig 2F), providing another line of evidence for the role of ATP hydrolysis by Rok1 in releasing Rrp5 from pre-40S subunits. Importantly, these experiments can readily distinguish between binding by the Rrp5_C7•Rok1 complex or binding by Rrp5_C7 or Rok1 alone, as each of these alone binds more weakly than the complex of the two (S2C Fig). Furthermore, this difference is likely an underestimate of the true affinity difference, as the lowest concentration of Rrp5•Rok1 in these experiments was 100 nM, necessary because lower concentrations did not produce enough Rrp5•Rok1 complex under these salt concentrations.

### ATP Hydrolysis by Rok1 Is Required for Release of Rrp5 from Pre-40S Ribosomes In Vivo

To test if ATP hydrolysis by Rok1 was required for release of Rrp5 from pre-40S subunits in vivo, a galactose-inducible/glucose-repressible promoter was introduced upstream of the *ROK1* gene in either an Rrp5-TAP or Enp1-TAP strain background. The resulting strains were transformed with plasmids harboring either wild-type (WT) or ATPase deficient (K172A or D280A) Rok1 under a constitutive promoter. Endogenous Rok1 was depleted in the presence of glucose, and ribosome biogenesis intermediates were captured via tandem affinity purification (TAP)-tag-mediated purification. If ATP hydrolysis by Rok1 were important for Rrp5 release from pre-40S subunits, we would expect that in cells with ATPase-deficient Rok1, Rrp5 would accumulate in pre-40S complexes and be depleted from pre-60S complexes relative to the WT cells.

**Fig 3:**
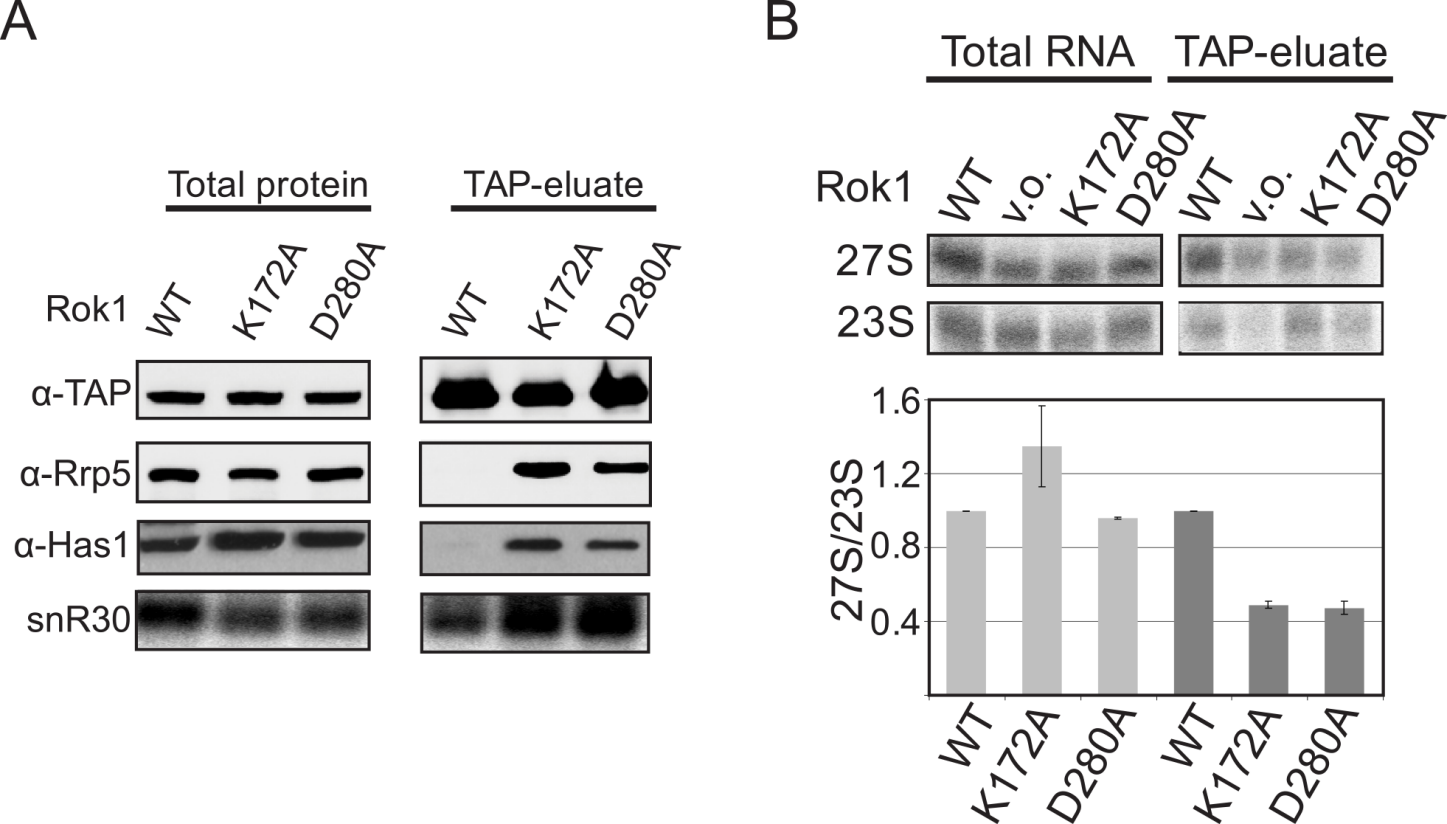
ATP-hydrolysis by Rok1 is required for Rrp5 release from pre-40S ribosomes in vivo. (A) Western and northern blot analysis of pre-40S ribosomes purified from yeast cells with Enp1-TAP reveals the accumulation of Rrp5, Has1, and snR30 in pre-40S complexes isolated from Rok1 ATPase-deficient cells relative to the WT cells. The TAP-antibody was used as a loading control. (B) Northern blot analysis of preribosomes captured with Rrp5-TAP in the presence of wild-type Rok1 (WT) or ATPase-deficient mutants of Rok1 (K172A and D280A) reveals decreased binding to pre-60S subunits (containing 27S pre-rRNAs) relative to pre-40S subunits (containing 23S pre-rRNA). The data from three biological replicates were quantitated and normalized such that in wild-type cells the 27S/23S levels are set to 1 and the mutants are expressed relative to the WT. The numerical data underlying this graph can be found in S1 Data.

To test this prediction, we assayed for copurification of Rrp5 with the 40S AF Enp1. Enp1 binds nascent 40S ribosomes relatively early but then remains bound throughout most maturation steps; thus, it is a reliable target for purifying different 40S assembly intermediates [30]. While no Rrp5 copurifies with Enp1-tagged nascent 40S subunits in cells containing wild-type Rok1, we observe efficient copurification of Rrp5 in cells containing ATPase-impaired Rok1 (Fig 3A). This observation supports our model that the ATPase activity of Rok1 is required for the release of Rrp5 from pre-40S complexes.

To further confirm that the ATPase activity of Rok1 regulated the binding of Rrp5 to nascent 40S and 60S subunits in vivo, and exclude possible indirect effects, we compared the levels of 40S and 60S precursors that copurify with Rrp5-TAP. Because A_2_ cleavage is impaired in cells with ATPase-deficient Rok1, leading to depletion of 20S rRNA (S3 Fig, [21]), we used 23S rRNA as a pre-40S marker and 27S rRNAs as pre-60S markers. Importantly, the levels of these RNAs are essentially unchanged in cells containing mutant Rok1 compared to WT Rok1 (Fig 3B and S3 Fig), but we observe a more than 50% decrease in the binding of pre-60S to Rrp5, while pre-40S are enriched (Fig 3B), supporting our model that Rok1 governs the release of Rrp5 from pre-40S subunits to allow for binding to pre-60S subunits. Nevertheless, it is important to note that these in vivo data alone would be consistent with a number of scenarios, as direct effects are hard to distinguish from indirect effects, as described in more detail for snR30 accumulation below.

### Rrp5 Binds to Rok1 via Multiple Domains

Mutation of the Walker A motif in Rok1 leads to a ~2-fold accumulation of snR30 on pre-40S ribosomes relative to wild-type Rok1 encoded on the same plasmid [22], leading to the suggestion that Rok1’s function in 40S assembly is to release snR30. Release of snR30 could be a second function of Rok1 in addition to the release of Rrp5 demonstrated herein. Alternatively, snR30 accumulation could be a secondary effect that arises (directly or indirectly) from blocked Rrp5 release. Importantly, mutation or depletion of Rok1 is not the only event that leads to snR30 accumulation in pre-40S ribosomes: both Has1 and Utp23 are also required for snR30 release from pre-40S ribosomes [23,24]. Interestingly, recent yeast two-hybrid screens indicate that Rrp5 and Has1 are part of a common protein-protein interaction network [31–33]. Furthermore, Has1 accumulates on pre-40S subunits when Rok1 is inactive, thus depleting it from solution (Fig 3A).

We therefore wanted to test if the snR30 accumulation in pre-40S observed upon Rok1 inactivation was a direct effect from Rok1 inactivation or instead due to the failure to release Rrp5. To distinguish between these possibilities, we wanted to produce an Rrp5 mutant that phenocopies the Rrp5 accumulation in pre-40S ribosomes observed upon Rok1 inactivation. We rationalized that a mutation that disrupts Rrp5•Rok1 interactions would produce such an effect, as Rrp5 would no longer be released in response to Rok1 ATP hydrolysis. To design such a mutant, we obtained insight into the structure of the Rrp•Rok1 complex using a combination of 3DEM, X-ray crystallography, and biochemical analyses as described below.

**Fig 4:**
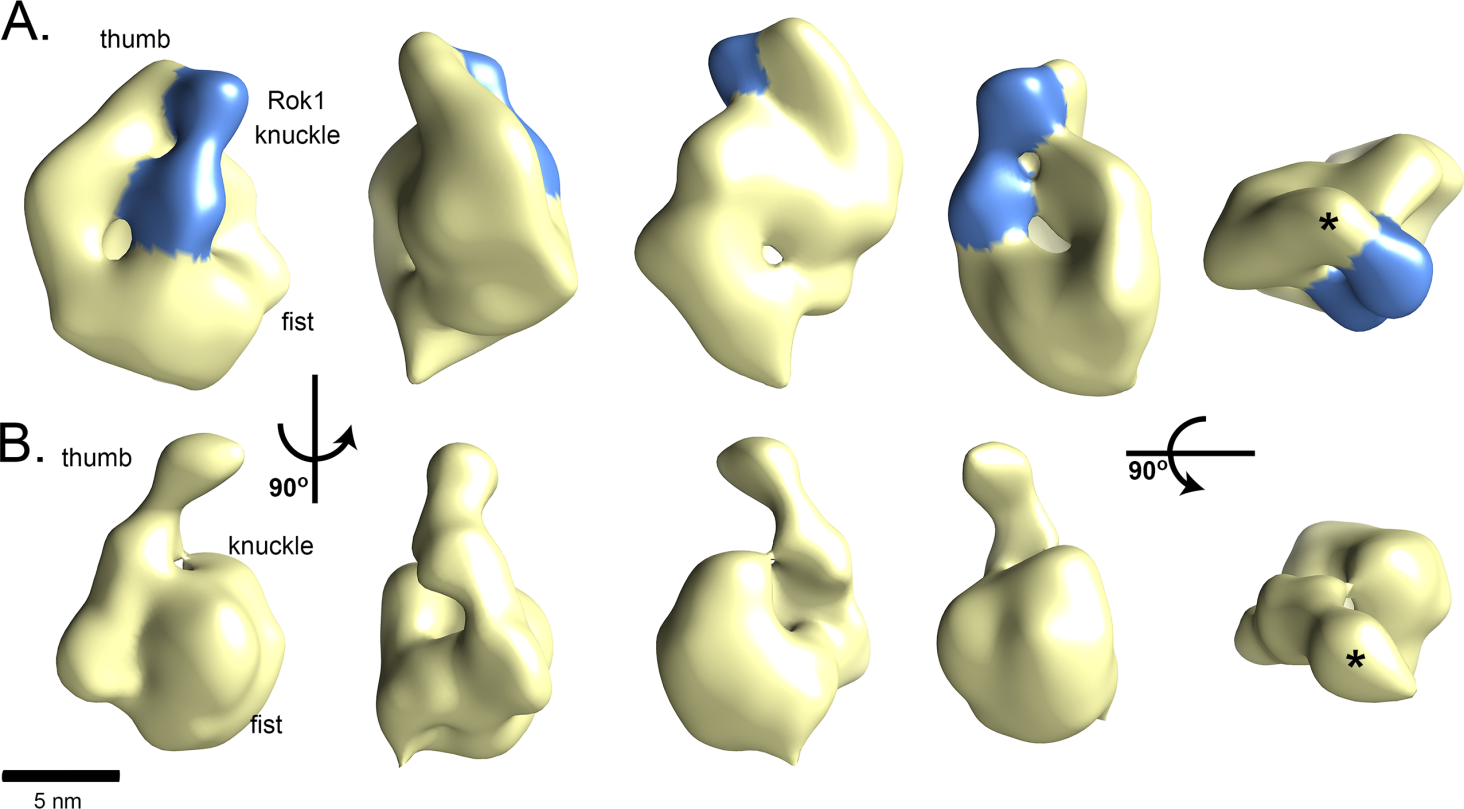
The structure of Rrp5 complexes. 3-D reconstructions of (A) Rrp5•Rok1 or (B) Rrp5•RNA. 90° rotations of Rrp5 complexes around its long axis show the curved, thumb-like projection and hollow fist-like body. The area corresponding to Rok1’s RecA motifs is shaded in blue. By Fourier shell correlation (FSC) = 0.5, the structures are each at about 2.8 nm resolution (S9A Fig). In the final column, the asterisk marks the tip of the TPR.

We used low-resolution single-particle negative-stain EM to gain insight into the architecture of the complex formed between Rok1 and Rrp5 (Rok1•Rrp5 complex, Fig 4A). 3-D reconstruction of the Rok1•Rrp5 density (Fig 4A, EM Database code 6654) shows a molecule with a round, hollow fist-like base and a slender thumb-like projection, with a bipartite density bridging the thumb to the fist and the knuckle. Contouring at 6.2 σ threshold represents a mass of 260 kDa that accounts for 193 kDa of Rrp5 and 64 kDa of Rok1.

To dissect which parts of this structure belong to Rrp5 and which to Rok1 and to identify the contact points between Rrp5 and Rok1, we imaged Rrp5 alone. Two-dimensional (2-D) reference-free alignment, classification, and averaging show that Rrp5 is a round fist with a projection, suggesting that the bridge between the knuckle and the thumb represents Rok1 (S4B Fig). However, further 3-D image analysis of these samples did not converge to a well-defined structure, suggesting that Rrp5 alone is structurally flexible. To stabilize its structure, we added an in vitro transcribed rRNA mimic to Rrp5 (S4C Fig). The RNA mimic we utilized comprises the ITS1 sequence, which forms the main binding site between Rrp5 and preribosomes (S1B Fig, [12,13]). 3-D reconstruction of the density observed for the complex between Rrp5 and RNA (Rrp5•RNA) (EM Database code 6655) shows an elongated molecule with a round, hollow fist-like base and a slender thumb-like projection (Fig 4B). 2-D alignment and classification and 3-D analysis by random conical tilt (RCT) confirm this assessment (S5 Fig). These data suggest strongly that the bridging density, observed only in the Rrp5•Rok1 structure, is contributed by Rok1. Consistently, this density can accommodate a DEAD-box protein in the open conformation, placing each of the RecA-like domains into its lobes (S6A Fig).

To identify the contacts between Rok1 and the thumb and the fist, we next set out to identify the location of the TPR domains in Rrp5. Previous genetic interactions suggested a role for the TPR domain in Rok1 binding [34]. Reference-free alignment, classification, and averaging show that RNA-bound Rrp5_N12 (lacking the TPR domain) is a fist without the thumb-like projection (S4D Fig), suggesting that the thumb contains the tandem TPR repeats. As observed for Rrp5 alone, further 3-D image analysis of Rrp5_N12•RNA did not converge to a well-defined structure.

**Fig 5:**
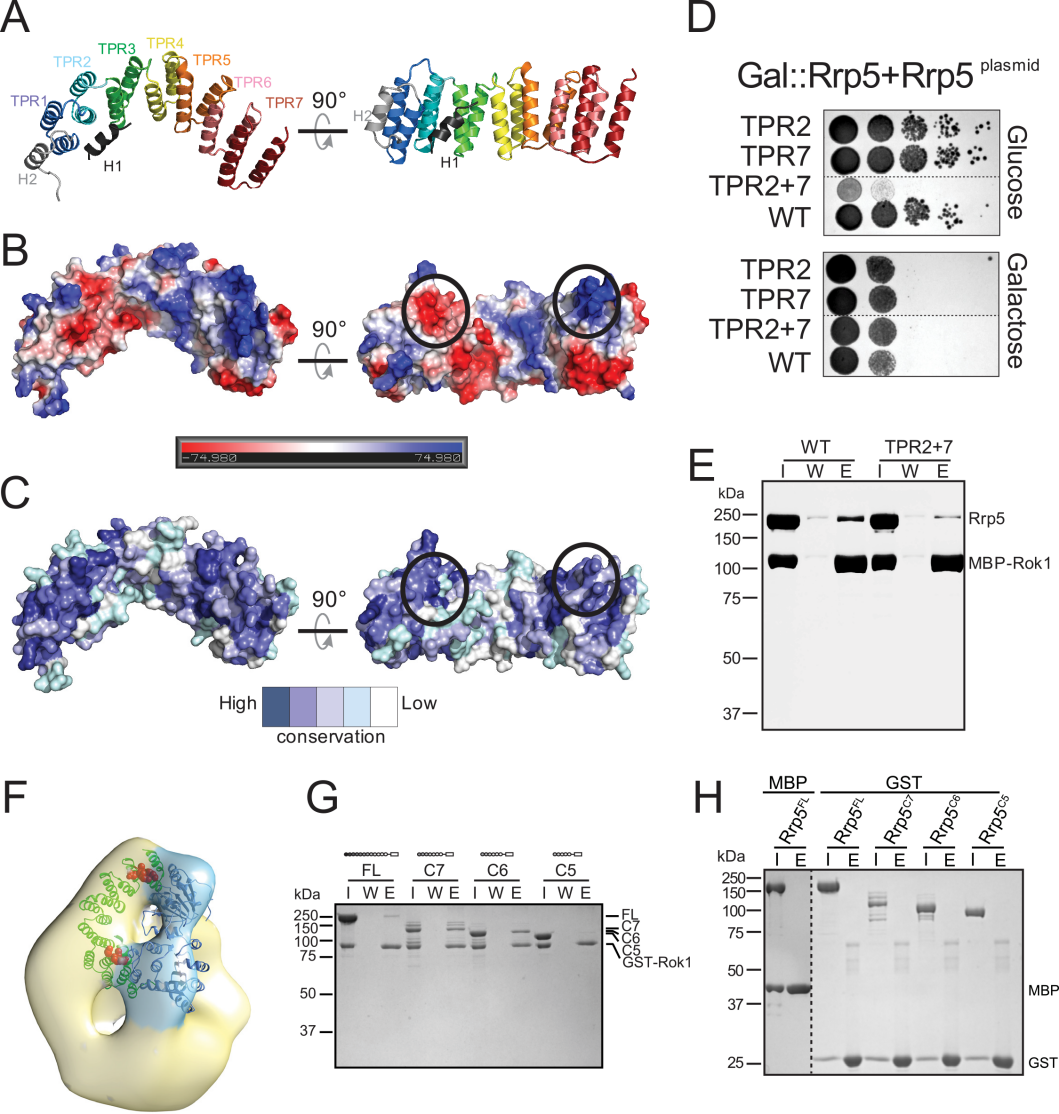
The structure of the TPR domain of Rrp5. (A) Cartoon representation of the crystal structure. (B) Surface charge distribution of the TPR domain of Rrp5. (C) Surface representation of amino acid conservation calculated using the ConSurf server [35] and displayed in PyMOL (The PyMOL Molecular Graphics System, Version 1.0r1 Schrödinger). The program ConSurf uses the provided sequence (*S*. *cerevisiae* Rrp5) to find the 150 closest homologs (lowest E-values) for generating a multiple sequence alignment. The organisms used for Rrp5-TPR can be found here: http://consurf.tau.ac.il/results/1463756501/query_final_homolougs.html. The conserved patches on the surface of the TPR motif that interact with Rok1 are marked in red. (D) Mutations in conserved charged areas of the TPR domain are deleterious in vivo. TPR2: E1509K/E1510K/E1512K and TPR7: K1686E/K1689E. (E) Mutations in conserved charged areas of the TPR domain weaken the Rok1•Rrp5 interaction in vitro. (F) Position of the TPR mutations in the 3-D reconstruction of the Rrp5•Rok1 complex. (G) Recombinant GST-Rok1 immobilized on glutathione (GSH) sepharose resin interacts with recombinant, purified Rrp5^FL^, Rrp5_C7, and Rrp5_C6, but not Rrp5_C5. (H) Maltose binding protein (MBP) or glutathione S-transferase (GST) alone does not bind to the Rrp5 fragments tested here. I, input; W, wash; E, elution.

To confirm that the thumb-like projection could contain the TPR motifs, we crystallized Rrp5_C2 and solved the structure by single wavelength anomalous dispersion (SAD) to a resolution of 2.7 Å (Fig 5A, S1 Table). The resulting structure (Protein Data Bank [PDB] code 5C9S) resolves 277 out of 646 residues in Rrp5_C2. Analysis of the crystal content by SDS-PAGE revealed that the protein was partially degraded, resulting in a fragment whose size is consistent with the TPR domain, which is resolved in the structure and is composed of 16 antiparallel α-helices, 14 of which (H3-H16) create seven helix-turn-helix units with the canonical features of TPR motifs (S7A–S7C Fig). The two helices of a unit are connected by a tight turn, and each unit is connected to the next by a variable linker. The most N-terminal TPR unit is connected to a helix (H2) in the region between the TPR and the S1 domains. Partial density for another helix of this region (H1, aa 1408–1420) is visible at the concave surface of the TPR domain. This helix is held in place via interactions with H3, H5, and H7 (S7D Fig). Importantly, the crystal structure can be docked into the thumb (S6B Fig), suggesting that the TPR domains in the thumb interact with Rok1.

Interestingly, two of the three contact points between Rok1 and Rrp5 are located near a conserved N-terminal acidic and C-terminal basic patch on the surface of the TPR motif, suggesting that these patches likely form part of the interface with Rok1 (Fig 5B and 5C). To test this prediction, we mutated surface-exposed conserved amino acids in these patches and tested these Rrp5 mutants for complementation of the growth defect observed upon growth of a Gal::Rrp5 strain in glucose. This screen identified two mutations, one in TPR helix 2 and the other in TPR helix 7, that, when combined, produce a severe growth phenotype in vivo (Fig 5D). Moreover, the double mutant, TRP2+7, is also defective for Rok1 binding in vitro (Fig 5E). These data demonstrate that two of the three Rok1•Rrp5 contacts are made with the TPR domain of Rrp5 (Fig 5F), consistent with previous genetic interactions [34]. Furthermore, these data also strongly support our interpretation of the Rrp5•Rok1 structure.

To identify the third contact between Rrp5 and Rok1, we used truncation analysis. While glutathione S-transferase (GST)-tagged Rok1 (GST-Rok1) bound to full-length Rrp5, Rrp5_C7, or Rrp5_C6, it did not bind Rrp5_C5, demonstrating that the sixth S1 domain contributes to the Rrp5•Rok1 interface (Fig 5G). Control experiments show that these interactions are specific to Rok1 and not the tag or the resin (Fig 5H). We also note that Rrp5_C7 binds GST-Rok1 more readily than Rrp5_FL. This could indicate that only a fraction of Rrp5_FL is active, perhaps not surprising for such a large protein. However, we also note that maltose binding protein (MBP)-tagged Rok1 (MBP-Rok1) bound Rrp5 more tightly than GST-Rok1 (Fig 5E and 5G). Similarly, GST-Rok1 did not bind Rrp5_C3, while MBP-Rrp5_C3 binds Rok1 well [14]. Thus, it appears that the GST-tag of Rok1 interferes with Rrp5 binding, perhaps because of the ability of GST to dimerize. Regardless, our biochemical data provide strong evidence for multiple contacts between Rok1 and Rrp5. These are formed by the TPR and the sixth S1 domain of Rrp5 (Fig 5), consistent with and supporting our interpretation of the Rrp5•Rok1 structure (Fig 4). Importantly, Rrp5 domains responsible for 40S binding are directly adjacent to those required for Rok1 binding (S1D Fig), readily rationalizing how Rok1 binding could affect Rrp5’s ability to bind to 40S ribosomes, via remodeling of the interactions with adjacent S1 domains.

### Accumulation of snR30 in Pre-40S Subunits upon Inactivation of Rok1 Is an Indirect Effect from Blocked Rrp5 Release

As described above, we wanted to distinguish between two alternative models to explain the observed snR30 accumulation in pre-40S ribosomes [22]: (i) a direct role for Rok1 in unwinding the snR30•18S rRNA helix or (ii) snR30 accumulation after Rok1 inactivation being a consequence of failure to release Rrp5. The TPR2+7 mutation in Rrp5 is defective in binding to Rok1 (Fig 5D and 5E). If the only role of Rok1’s ATPase activity is to release Rrp5 from pre-40S subunits, and the pre-40S accumulation of Has1 and snR30 upon Rok1 inactivation is an indirect effect from Rrp5 accumulation, then we would expect the TPR2+7 mutations in Rrp5 to phenocopy the ATPase-inactive Rok1 mutants. In contrast, if the Rok1 ATPase activity has a second function that directly leads to release of Has1 and/or snR30, then Has1 and snR30 should not accumulate in pre-40S ribosomes when Rrp5 is mutated, as Rok1 is active in these strains.

**Fig 6:**
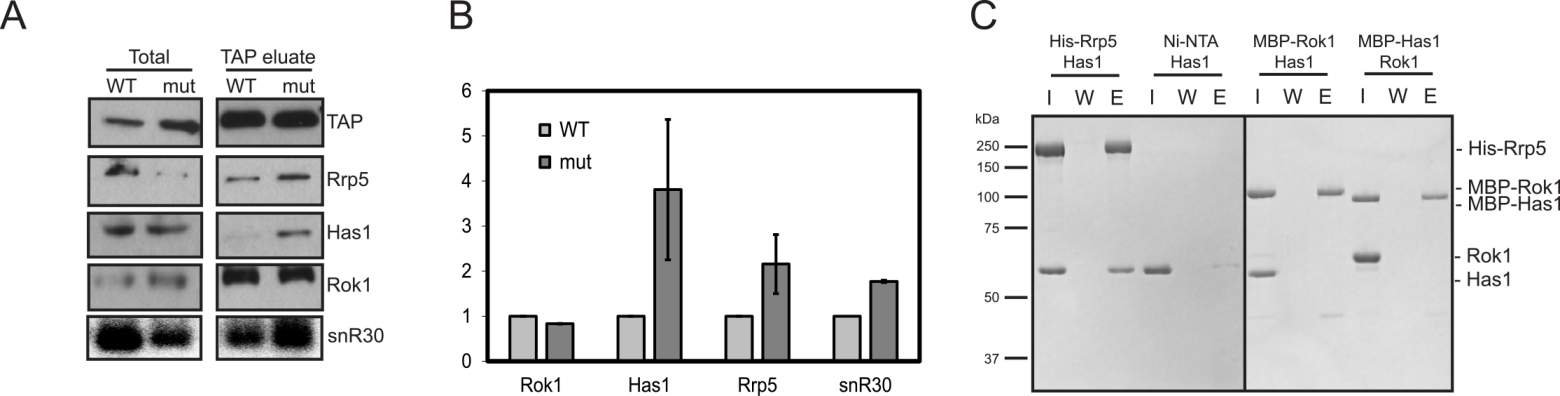
Has1 and snR30 accumulate in pre-40S subunits when the Rok1•Rrp5 interaction is disrupted. (A) Western and northern blot analysis of pre-40S ribosomes purified with Enp1-TAP from yeast cells expressing wild-type Rrp5 or TPR2+7-mutant Rrp5. The TAP-antibody was used as a loading control. (B) Quantitation of two biological replicates of data on the left. The difference in copurification of the named AFs with mutant and wild-type Rrp5 is shown. The numerical data underlying this graph can be found in S1 Data. (C) Recombinant Has1 binds recombinant His_6_-Rrp5, but not recombinant MBP-Rok1, and recombinant MBP-Has1 does not bind recombinant Rok1. Has1 alone does not bind to the Ni beads.

Enp1-TAP-purification of pre-40S subunits from yeast cells expressing the Rrp5_TPR2+7 mutant demonstrates that Rrp5 accumulates in pre-40S subunits in this mutant relative to wild-type Rrp5 (Fig 6A and 6B), as expected from the disrupted Rok1•Rrp5 communication in these mutants, which blocks ATPase-dependent release of Rrp5. Importantly, our data also show that Rok1 recruitment to pre-40S is not affected by these mutants, ruling out the absence of Rok1 as causative of these effects. Importantly, the data also show that Has1 and snR30 are accumulated in these precursors, demonstrating that it is not the ATPase activity of Rok1 that is required for Rrp5, Has1, and snR30 release from pre-40S subunits. Instead, these data show that communication between Rok1 and Rrp5 is required for Rrp5, Has1, and snR30 release.

As described above, Has1 and Rrp5 were recently shown to be part of a common protein-protein interaction network [31–33]. Furthermore, Has1 is required for snR30 release [23]. To test if Rrp5 and Has1 directly bound each other, we cloned the HAS1 gene from yeast, purified recombinant Has1 protein from *Escherichia coli*, and tested it for binding to His_6_-Rrp5 on Ni-beads. Retention of Has1 on Ni-beads in the presence but not the absence of Rrp5 (Fig 6C, left) demonstrates that Has1 binds directly to Rrp5. In contrast, no binding between Has1 and MBP-Rok1 or MBP-Has1 and Rok1 was observed (Fig 6C, right). These data suggest that the observed accumulation of snR30 in pre-40S ribosomes observed upon Rok1 inactivation is a result of blocked Rrp5 release, which leads to accumulation of Has1 in pre-40S ribosomes, due to a direct interaction between these two proteins. The resulting loss of free Has1 then mediates (directly or indirectly) the accumulation of snR30.

## Discussion

### ATP Hydrolysis by Rok1 Releases Rrp5 from Pre-40S Subunits

Rok1 is a member of the DEAD-box class of ATPases. These ubiquitous regulators of RNA biology are often referred to as RNA helicases because of their structural similarity to the RecA-type DNA helicases. Nevertheless, DEAD-box proteins are not processive helicases [19,20], and only a subset of family members have demonstrated helix dissociation activity. Instead, their characterized functions include duplex annealing, RNA protein complex disruption, and ATP-dependent RNA binding [15–18]. Rok1 does not appear to have substantial helicase activity [14,25] but efficiently anneals two strands of pre-rRNA in an ATP-independent manner [14]. However, mutation of the residues required for ATP binding and hydrolysis is lethal [21] and blocks pre-18S rRNA processing (S3 Fig), indicating an additional role for Rok1 in 40S ribosome assembly.

Rrp5 functions in both 40S and 60S biogenesis, owing to its interactions with ITS1, 18S, and 25S rRNAs and 40S and 60S AFs [12,34,36–38]. Upon rRNA cleavage within ITS1, Rrp5 interactions with 40S pre-rRNA must be disrupted while it remains bound to the maturing 60S [11]. Here, we use a combination of in vitro and in vivo experiments to provide evidence that ATP hydrolysis by Rok1 is responsible for release of Rrp5 from pre-40S subunits, thereby enabling Rrp5’s interaction with pre-60S subunits. Thus, Rok1 orchestrates the assembly of 40S and 60S ribosomal subunits by regulating the release of Rrp5 from nascent 40S subunits, thereby allowing its binding to pre-60S subunits. The fact that this ADP-dependent release can be reconstituted in a simple in vitro system, consisting only of Rok1, Rrp5, nucleotide, and 40S subunits, or even rRNA mimics, demonstrates that Rok1 is directly responsible for Rrp5 release, as opposed to there being indirect effects mediated by other factors. Structural and biochemical analyses of the Rrp5•Rok1 complex suggest that Rok1 forms multiple contacts with regions in Rrp5, which are adjacent to those required for 40S binding, thus explaining the allostery in Rok1, ATP, and 40S binding.

In contrast, our data also suggest that the previously observed accumulation of snR30 upon Rok1 inactivation [22] is an indirect effect that arises from blocked Rrp5 release. Importantly, snR30 accumulation is also observed when Rok1 is active, but communication between Rok1 and Rrp5 is disrupted by mutations in Rrp5’s TPR domain. Furthermore, our data also suggest that this effect is mediated by Has1, which is required for snR30 release from pre-40S subunits [23], also accumulates in pre-40S subunits upon Rok1 inactivation or disruption of Rok1•Rrp5 communication, and binds directly to Rrp5.

### Model for Rok1 Function in Ribosome Maturation

**Fig 7:**
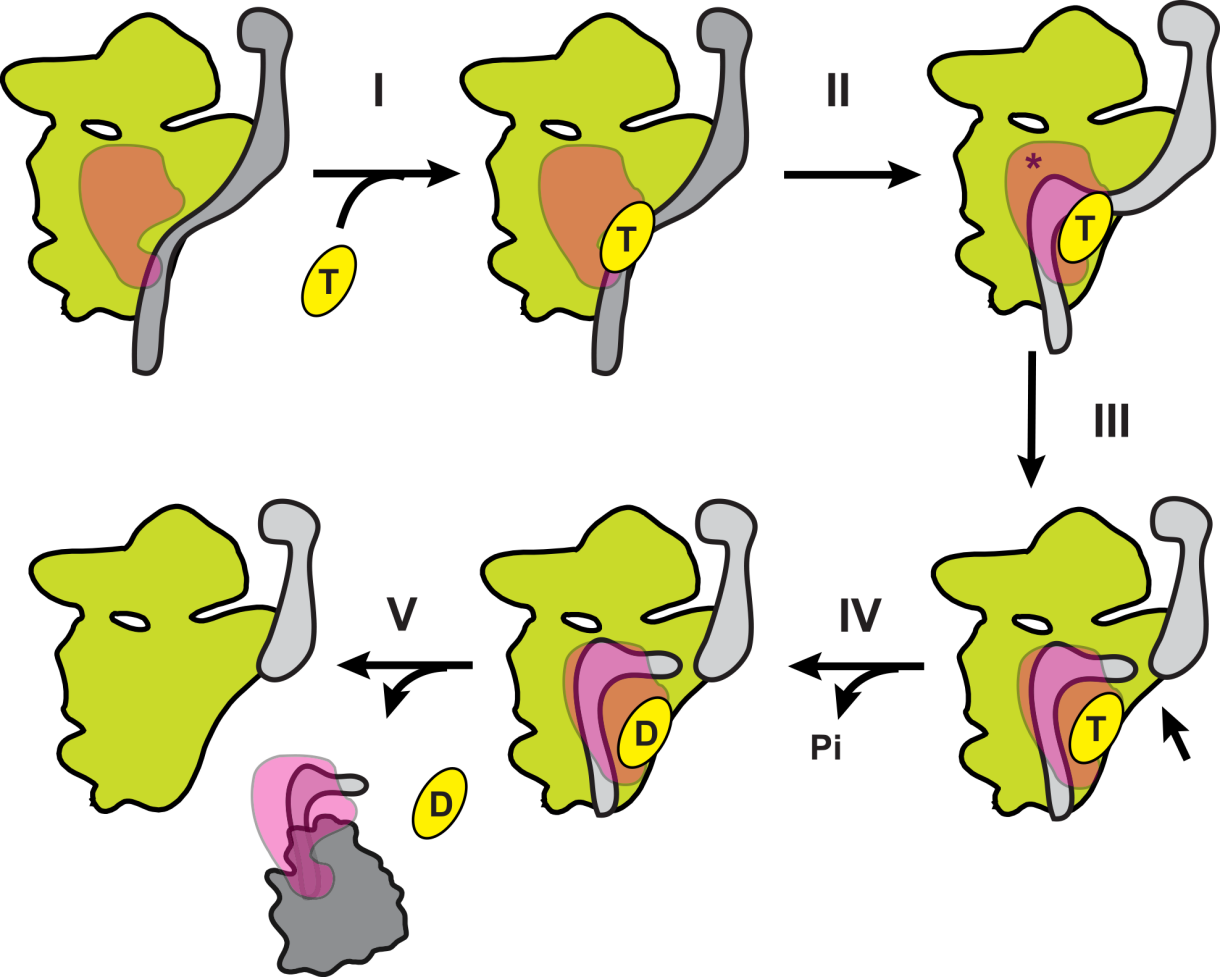
Speculative model for Rrp5•Rok1 function during 40S ribosome maturation. Rrp5 (in magenta) is bound to pre-40S subunits (green) and interacts with ITS1 (grey). Rok1 (in yellow) is recruited to pre-40S subunits (I),and then forms the inhibitory duplex to block premature 3′-end formation (II, different secondary structure shown in lighter grey, indicated by asterisk). After cleavage in ITS1 (III, marked by arrow), ATP (T) is hydrolyzed to ADP (D, IV), leading to dissociation of Rrp5•Rok1 from pre-40S subunits (V).

Based on previously reported data and the data herein, we propose a model for Rok1’s functional cycle during 40S ribosome assembly (Fig 7). Previous data indicate that Rrp5 binds relatively early to assembling pre-40S subunits [9,26,39]. Once Rrp5 is loaded onto pre-40S subunits, it helps recruit Rok1 (step I, Fig 7, [10,14]). Although Rok1 does not have substantial unwinding activity, it effectively anneals duplexes and, in the presence of Rrp5, specifically promotes formation of a duplex between ITS1 and the top of the decoding site helix, H44 (step II, [14,25]). This duplex inhibits premature formation of the 18S rRNA 3′-end [40]. Interestingly, recent cross-linking data position Rok1 adjacent to H44 (S8 Fig, [41]), as predicted from a role for Rok1 in establishment of this structure. After cleavage within ITS1 (step III), Rok1 ATPase activity must be triggered through an unknown mechanism (step IV), thus allowing for dissociation of Rrp5 from pre-40S subunits (step V), while preserving the stronger interactions with the regions in ITS1 that will remain part of pre-60S subunits [7,11,12]. Notably, much work remains to clarify aspects of the Rrp5/Rok1 functional cycle beyond the ATPase-dependent release of Rrp5/Rok1.

### DEAD-box Proteins as ATP-Dependent Molecular Switches

Our data strongly suggest that no mechanical work powers the release of Rrp5•Rok1 from pre-40S ribosomes and instead indicate that conformational changes between the ATP-and ADP-bound forms of Rok1•Rrp5 are responsible for the release. This is reminiscent of the ATPase-dependent release of the exon-junction complex (EJC) from mRNAs. In that case, the DEAD-box protein eIF4AIII cooperatively binds the ATP-analog AMPPNP, RNA, and the EJC-components Magoh, Y14, and MLN51, clamping the EJC onto the RNA in the presence of ATP [42]. In contrast, large changes in eIF4AIII in the ADP bound form lead to disassembly of the entire complex after ATP hydrolysis and dissociation of the phosphate [43–45]. This conformational switch is analogous to the role of ADP-bound Rok1 in Rrp5 release from the 40S rRNA that we demonstrate here.

## Materials and Methods

### Cloning and Mutagenesis

Rrp5_C8, Rrp5_C7, Rrp5_C6, Rrp5_C5, and Rrp5_C2 were cloned into pGEX-6-P3 (GE Healthcare) containing an N-terminal GST-tag. Rrp5_N12 was cloned into a modified pET23b vector with an N-terminal 6×His-tag followed by a TEV cleavage site. Has1 was cloned into pSV272, for expression as a TEV-cleavable His_6_-MBP fusion protein.

Rok1, Rrp5-FL, Rrp5_N12, Rrp5_N9, Rrp5_C8, and Rrp5_C7 were cloned into pRS416-TEF for yeast expression.

All mutagenesis was carried out according to the Quikchange protocol.

### Protein Expression and Purification

All proteins were expressed in *E*. *coli* Rosetta2 (DE3) cells (Novagen). Cells were grown at 37°C to OD_600_ of 0.6 in 2×YT media supplemented with the appropriate antibiotic and then transferred to 18°C. Protein expression was induced by addition of 0.3 mM or 1 mM IPTG for pGEX-6-P3 or pET23/pSV272 harboring cells, respectively. Bacterial cultures were harvested after 18 h.

Rrp5_C8, Rrp5_C7, Rrp5_C6, Rrp5_C5, and Rrp5_C2 were purified using GSH sepharose resin (GE Healthcare) in GST-binding buffer (300 mM NaCl, 50 mM HEPES/NaOH [pH 7.5], 10% glycerol, and 2 mM β-mercaptoethanol [βME]). After washing the beads extensively with the same buffer, protein was eluted in GST-binding buffer supplemented with 20 mM reduced glutathione. The GST-tag was cleaved off using PreScission protease (GE Healthcare) while dialyzing overnight in 200 mM NaCl, 30 mM HEPES/NaOH (pH 7.5), 10% (w/v) glycerol, and 2 mM βME. GST and the protease were removed, and the protein was further purified using a MonoQ ion exchange column (GE Healthcare) equilibrated with the dialysis buffer. The protein was eluted with a high salt gradient (1 M NaCl, 30 mM HEPES/NaOH, 10% glycerol, and 2 mM βME) over ten column volumes. The purified protein was further polished using Superdex S-200 gel filtration column (GE Healthcare) equilibrated in 200 mM NaCl, 20 mM HEPES/NaOH, 5% glycerol, and 1 mM DTT.

Rrp5_N12 was purified over Ni-NTA affinity resin according to the manufacturer’s recommendation (Qiagen). The eluate was dialyzed for 4 h into 200 mM NaCl, 30 mM Tris/HCl (pH 8.5), 5% glycerol, and 2 mM βME and applied to a MonoQ column in the same buffer. Rrp5_N12 was eluted with increasing salt concentration and further purified using a Superdex S-200 column in 200 mM NaCl, 20 mM Tris/HCl (pH 8.5), 5% glycerol, and 1 mM DTT.

To prepare selenomethionine-substituted Rrp5_C2, Rosetta2 (DE3) cells were grown in a minimal media supplemented with selenomethionine [46]. Protein was purified as described before [12].

Has1 was purified over Ni-NTA resin (Qiagen) according to the manufacturer’s recommendation. The eluate was dialyzed overnight against 150 mM NaCl, 25 mM KH_2_PO_4_ (pH 7.5), 5% glycerol, and 2 mM βME and applied to a MonoS column in the same buffer. Has1 was eluted with increasing salt concentration and further purified using a Superdex S-200 column in 200 mM NaCl, 20 mM HEPES/NaOH (pH 7.5), 5% glycerol, and 1 mM DTT.

Rrp5-FL and Rok1 were purified as previously described [12,14].

### Yeast Strains and Plasmids

The *S*. *cerevisiae* strains and plasmids used in this study are listed in S2 Table and S3 Table, respectively. Yeast strains were generated using standard recombination techniques and verified by western blotting and/or colony PCR.

### Protein-Protein Interaction Studies

Ten μM of FL Rrp5 or Rrp5_TPR2+7 were mixed with 15 μM MBP-Rok1 (or MBP) in 150 mM NaCl and 30 mM HEPES/NaOH (pH 7.5), 5% glycerol, and preincubated on ice for 15 min before addition of 25 μl of equilibrated amylose resin (New England BioLabs). The mixture was incubated for 30 min at 4°C, the flow-through was collected, the resin was washed, and proteins were eluted with binding buffer supplemented with 20 mM maltose.

For binding experiments with different Rrp5 fragments, 5 μM FL Rrp5 or Rrp5 fragments were mixed with 3 μM GST-Rok1 (or GST) in 150 mM NaCl and 30 mM HEPES/NaOH (pH 7.5), 5% glycerol, and preincubated on ice for 15 min before addition of 25 μl of equilibrated GSH sepharose resin (GE Healthcare). The mixture was incubated for 30 min at 4°C, the flow-through was collected, the resin was washed, and proteins were eluted with binding buffer supplemented with 20 mM reduced glutathione.

For binding experiments with recombinant Has1, or MBP-Has1, 5 μM Has1 was mixed with 3 μM His-Rrp5 or GST-Rok1 in the pull-down buffer. Alternatively, 5 μM Rok1 was mixed with 3 μM MBP-Has1 in the same buffer and preincubated on ice for 15 min before addition of 25 μl of equilibrated Ni-NTA or GSH sepharose resin. The mixture was incubated for 30 min at 4°C, the flow-through was collected, the resin washed, and the proteins were eluted with binding buffer supplemented with 300 mM imidazole or 20 mM reduced glutathione.

### In Vitro 40S Ribosome Binding Studies by Gradient

Two hundred and forty nM of recombinant Rrp5 (or Rrp5 fragments) and mature 40S ribosomes were mixed with 2 μM recombinant Rok1 and 4 mM Mg•AMPPCP in ribosome binding buffer (200 mM KOAc, 20 mM HEPES/KOH [pH 7.4], 2.5 mM MgOAc and 2 mM DTT, 0.1 mg/ml heparin and 0.5 μl of RNasin [NEB]). After incubating for 15 min at 25°C and 10 min on ice, the complexes were layered on top of 11 mL 5%–20% glycerol gradients in gradient buffer (100 mM NaCl, 50 mM HEPES/NaOH, 10 mM MgCl_2_) and spun in a SW41 rotor (Beckman) at 40,000 rpm for 4 h at 4°C. The gradients were fractionated and the proteins were analyzed by SDS-PAGE followed by western blotting.

### In Vitro 40S Ribosome Binding Studies by Pelleting

Two hundred nM of recombinant Rrp5 or Rrp5 fragments was mixed with either buffer or 240 nM mature 40S ribosomes, purified as described [47], in ribosome binding buffer. Mixtures were incubated for 15 min at 25°C and 10 min on ice and layered on top of 400 μl sucrose cushion (ribosome binding buffer + 20% sucrose (w/v)] and spun for 2.5 h at 400,000 × g at 4°C in a TLA100.1 rotor (Beckman). The pellets were dissolved in SDS-loading dye. Supernatants were precipitated using trichloroacetic acid.

### In Vivo Preribosome Binding Assay

Yeast cells in which the endogenous *RRP5* gene is under a galactose inducible/glucose repressible promoter were grown in YPD for 20 h to deplete endogenous Rrp5 while different plasmid-encoded fragments of Rrp5 were constitutively expressed. Lysates were prepared from these cells and fractionated on a 5%–50% sucrose gradient as previously described [48,49]. The position of Rrp5 on these gradients was detected by western blot using an antibody raised against Rrp5.

### In Vitro Rrp5 Release Assay

Two-hundred and forty nM of recombinant Rrp5_C8 and mature 40S ribosomes were mixed with 2 μM recombinant Rok1 and 400 μM ADP or 4 mM AMPPCP in ribosome binding buffer supplemented with additional 400 μM or 4 mM MgOAc for ADP and AMPPCP, respectively. After incubating for 15 min at 25°C and 10 min on ice, the complexes were layered on top of 11 mL 5%–20% glycerol gradients in gradient buffer and spun in a SW41 rotor (Beckman) at 40,000 rpm for 4 h at 4°C. The gradients were fractionated and the proteins were analyzed by SDS-PAGE followed by western blotting. Quantitation was performed using Quantity One (BioRad). For control experiments, either Rrp5 or Rok1 was omitted. Rrp5_C8 was used, as it was easier to purify and more stable in the freezer than Rrp5-FL.

### In Vitro RNA•Protein Gel-Shift Assay

rRNA was folded in the presence of 10 mM Mg^2+^ as described [50]. Prefolded rRNA and recombinant Rrp5_C7, Rok1, or Rrp5_C7•Rok1 (Rrp5_C7:Rok1 1:1.1) were incubated in 200 mM KCl, 10 mM MgCl_2_, and 40 mM HEPES/KOH (pH 7.5) supplemented with either 10 mM Mg•AMPPCP or 1 mM Mg•ADP for 12 min at 25°C before being loaded on a 6% acrylamide/THEM (Tris, HEPES, EDTA at pH 7.5, MgCl_2_) gel [51] for 2 h at 4°C. Protein-bound and unbound fractions were quantified using phosphoimager software, and data were fit to a single binding isotherm using Kaleidagraph (Synergy Software).

### TAP Tag Purification and Protein/RNA Analysis

To test the role of Rok1 inactivation for Rrp5 retention in pre-40S or pre-60S subunits, yeast cells containing the ROK1 gene under the galactose-inducible/glucose-repressible Gal10 promoter were supplemented with plasmids encoding wild-type Rok1, or the indicated Rok1 mutants under the constitutive TEF promoter, and then grown for 20 h in YPD at 30°C. To probe the role of the Rrp5-Rok1 interaction for Rrp5, Has1, and snR30 release, yeast cells with the *RRP5* gene under a galactose-inducible/glucose-repressible promoter and supplemented with a plasmid encoding wild-type or TPR-mutant Rrp5 were grown for 20 h in YPD. TAP purification was carried out as described previously [52], and the eluates were probed by northern blotting for 23S rRNA (probe B: GCTCTCATGCTCTTGCC) and 27S rRNAs (probe 5.8S: CTGCGTTCTTGATCGATGCG) or by Western blotting against Rrp5 [14], Rok1 [14], TAP (Open Biosystems), and Has1 [53].

### Preparation of Rrp5 Complexes for EM Image Analysis

To assemble Rrp5•RNA complexes, 50 pmol of H45-3’ITS1 RNA was folded as previously described [50], equilibrated to 20°C, added to 100 pmol of Rrp5 or Rrp5_N12 protein (300 mM NaCl, 10 mM MgCl_2_, 30 mM MES, pH 6.7), and incubated at 20°C for 2 h. To assemble Rrp5•Rok1, 100 pmol of Rok1 was incubated with Rrp5 under identical conditions. Immediately after assembly, complexes were loaded onto a 5%–16% v/v glycerol and 0.01%–2% v/v glutaraldehyde GraFix gradient [54]. Apo Rrp5 and Rrp5_N12 were loaded directly onto the gradients. Gradients were centrifuged at 39,000 rpm at 4°C for 18 h in a SW41 rotor (Beckman). After fractionation, the fixation reaction was stopped by addition of glycine to a final concentration of 80 mM. Five μl of the different Rrp5 complexes was applied to glow discharged continuous thin carbon films. After the sample was blotted, 1.03 μm fiducial markers (Ted Pella) were added, and the grid was negatively stained with 2% (w/v) uranyl formate for 2 min.

### EM Data Collection and Processing

Tilted pair images of Rrp5 complexes were collected with an angle of 45° on a Titan-Krios TEM operated at 120 kV and at 59,000 times magnification on an UltraScan 4000 camera (Gatan), running the Leginon package for automated data acquisition [55,56]. Tilt pairs were handpicked, and the flat particles were aligned, classified, and averaged in Xmipp [57,58], as implemented in Appion [59]. Back projections of the individual class averages were calculated in SPIDER [60], which is implemented in Appion [59].

Flat images of Rrp5•RNA, Rrp5•Rok1, Rrp5, and Rrp5_N12 were collected under similar imaging conditions. Flat datasets were picked using the DoG picking algorithm in Appion [61]. Rrp5•RNA complexes were refined against the RCT starting model using projection matching in EMAN (S5 Fig) [62], with two independent datasets of 8,500 particles (S9 Fig). After a round of projection matching, each set of particles in each reference-based class average was subjected to further classification using the CAS command in SPIDER [60]; only the best 90% of the particles based on correlation to the projections were used for reconstruction. Both datasets converged to a similar structure, with a resolution of 29 Å resolution for Rrp5•RNA, judging by the Fourier shell correlation (FSC) of the two independently refined models at 0.5 (S9A Fig). To further validate the structure, we also used a 5 nm diameter ellipsoid as a starting model and refined the Rrp5 data as we did using the RCT-derived starting model (S9C Fig). The resulting structure is similar, with an equivalent resolution to the RCT-derived structure. In the final round of alignment, all particles were refined against the final half-dataset model, but only the best 50% of the particles were used in the final reconstruction. Including a higher percent of particles did not improve the resolution.

To obtain a reconstruction of the Rrp5•Rok1 complex, 10,500 particles were selected using the DoG algorithm in Appion and refined against the RCT starting model with the same iterative projection matching/classification algorithm described above. Eighty percent of the best particles were used in the final reconstruction, and using more of the data did not improve the resolution (30 Å resolution judging by FSC = 0.5).

Reference-free 2-D analysis of apo-Rrp5 and Rrp5_N12 provided comparable class averages to those from the larger complex, but refinement in 3-D did not coalesce to a single structure for either dataset, suggesting heterogeneity.

### Crystallization, Data Collection, and Structure Determination

Crystals of SeMet-Rrp5_C2 were grown at 20°C in sitting drop vapor diffusion plates by mixing 200 nl of crystallization solution (100 mM MES [pH 6.4], 32% PEG550 MME) and 200 nl of protein solution (at a concentration of 10 mg/ml in gel filtration buffer). Rrp5_C2 crystals were grown in 0.2 M ammonium tartrate, 20% PEG3350 reservoir. Crystals were cryoprotected by soaking in a solution containing 80% reservoir and 20% glycerol and flash frozen in liquid nitrogen before data collection.

SAD and native datasets were collected at 100 K on beamlines 21-ID-G at the Advanced Photon Source (APS) and 7–1 at Stanford Synchrotron Radiation Lightsource (SSRL), respectively. Data were indexed and integrated with iMosflm [63] and subsequently scaled using AIMLESS [64] from the CCP4 package [65]. The crystal structure of Rrp5_C2 was solved by SAD using phases from the Se-Met dataset at the peak wavelength calculated using the phasing module Autosol from the PHENIX program package [66,67]. Four out of seven heavy atom sites were identified unambiguously. Further density modification and model building were performed using the Autobuild module in the Phenix package [68]. The obtained partial model was manually completed in Coot [69] and used in Phaser [70] as a molecular replacement search model for the native dataset. This model was subsequently subjected to iterative steps of refinement in Phenix and manual model building in Coot.

## Supporting Information

S1 Data Contains the raw data for the quantifications in this work. (XLSX)

S1 Fig RNA and protein constructs used in this work. (A) Simplified rRNA processing scheme. (B) Schematic of H45-ITS1 and H44-A2 rRNAs used for EM analysis and protein•RNA binding experiments, respectively. (C) Domain architecture of Rrp5 and nomenclature for the fragments used throughout the text. (D) Summary of 40S and Rok1 interaction studies.
(EPS)

S2 Fig The nucleotide state of Rok1 does not affect Rrp5 binding but does affect RNA binding. (A) Protein-protein interaction assays in the absence of nucleotide and in the presence of 1 mM AMPPCP or 0.1 mM ADP. (B) Replicate of the data in Fig 2A. (C) Electrophoretic mobility shift assay of Rok1•AMPPCP (○), Rok1•ADP (□), or Rrp5_C7 (◇) binding to H44-A_2_. To facilitate comparison, the data for Rrp5_C7•Rok1•AMPPCP (●) from Fig 2F are replotted here. Binding data were fit with a single binding isotherm and yield *K*_1/2_ values for Rrp5_C7, Rok1•AMPPCP, and Rok1•ADP of 260 ± 122 nM, 201 ± 77 nM, and 197 ± 47 nM, respectively. The *K*_1/2_ value for Rrp5_C7•Rok1•AMPPCP is 57 ± 7 nM. *K*_1/2_ values were obtained from two or more replicate experiments. The numerical data underlying this graph can be found in S1 Data.
(EPS)

S3 Fig ATPase-deficient Rok1 blocks the early steps of pre-18S processing. Total RNA from Gal::Rok1;Rrp5TAP strains expressing either wild-type (WT) or two ATPase-deficient mutants of Rok1 (K172A and D280A) was analyzed by northern blot. Endogenous Rok1 was depleted by growth in YPD for 20 h. V.o., empty vector.
(EPS)

S4 Fig Alternative Rrp5 constructs localize the TPR motifs to the thumb-like projection. (A) Field of view of Rrp5•Rok1 and the reference-free class averages of the particles (inset) show particles with a fist-like base and a thumb-like slender extension. (B) Field of view of apo Rrp5 shows that without bound RNA, the protein is quite heterogeneous. Nonetheless, reference-free class averages of the particles (inset) show similar views to those seen in Rrp5•RNA. (C) Field of view of Rrp5•RNA shows particles similar to Rrp5•Rok1 in panel A. However, Rrp5•Rok1 has a slightly larger fist and additional density bridging the thumb, marked with an asterisk. (D) Field of view of Rrp5_N12, lacking the TPR motif, also shows a heterogeneous particle that could be aligned and averaged in 2-D. Reference-free class averaging shows a round hollow molecule with no thumb-like projection (inset). All scale bars are at 15 nm.
(EPS)

S5 Fig Model building of Rrp5•RNA by random conical tilt (RCT). Column 1: Reference-free class averages of negatively stained Rrp5•RNA show a round, fist-like core with a thumb-like projection. Column 2: The reprojection of the averaged RCT volume shows similarity to the averages in column 1. This model was used as the starting model for refining both the Rrp5•RNA and Rrp5•Rok1 structures. Column 3: The corresponding reprojection of the final Rrp5•RNA structure.
(TIF)

S6 Fig Interpretation of the Rrp5•Rok1 3D structure model. (A) Rok1 binds in a cleft between the thumb and the fist of Rrp5. Rrp5•Rok1 has a two-lobed density between the thumb and the fist. This density is reminiscent of “open” DEAD-box binding proteins that have been well characterized by X-ray crystallography. Apo eIF4AIII (PDB code 2HXY, [44]), a homologous DEAD-box protein that is the central molecule of the exon-junction complex (EJC), was docked manually into the two-lobed density in chimera. As docked, the N-terminus of the protein is placed closer to the fist. (B) Rrp5-TPR motifs fit into the thumb of the 3DEM structure. Rrp5_C2 was crystallized and its structure solved, revealing the shape of the C-terminal TPR motifs. When compared to the 3DEM structure of negatively stained Rrp5•RNA, the shape and size of the crystallized molecule fits well into the thumb-like projection. The S-ray structure of the TPR motifs was docked manually using Chimera.
(EPS)

S7 Fig The organization of the Rrp5 TPR motifs. (A) All seven TPR motifs are aligned based on their structure. Most of them are composed of two 30-amino-acid-long α-helices, which are connected to the next TPR motif via a 4-amino-acid-long loop. Helix A in TPR 2 and 7 are longer than the others by four residues. Helix B in TPR 3 is one amino acid longer than the others. (B) Structure-based sequence alignment of seven TPRs shows that they contain the TPR-specific sequence motifs [8]. However, position 7 is unique, as it is often occupied by an aromatic side chain, which interacts with the side chain at position 24. (C) Interaction between the aromatic (or bulky) side chains at positions 7 and 24 helps the consecutive TPR motifs to align perfectly relative to each other (as marked by dashed ovals) in Rrp5 structure. (D) Close-up of the interactions between H1 and the TPR domain. Hydrogen bonding interactions between the side chains of Thr1410 and Asn1480 (H3) and Gln1416 and Asn1522 (H5), as well as the carbonyl oxygen of Gln1416 and the side chain Lys1552 (H7), hold H1 in place. The distances measured between these side chains are 2.8 Å, 3.4 Å, and 2.6 Å, respectively.
(EPS)

S8 Fig The Rok1 binding site near H44. The Rok1 crosslink sites are shown in cyan. H27, which cross-links to both Rok1 and Rrp5, is shown in red. The structure of the helicase core domain of eIF4III (PDB code 2HXY, [44]) is shown next to the cluster of binding sites to demonstrate that it fits the site well.
(EPS)

S9 Fig Validation of structures. (A) Fourier shell correlation (FSC) analysis of both structures, each refined and reconstructed as two independent half sets. At correlation of 0.5, both are at about 2.8 nm resolution. (B) Two half sets of Rrp5•RNA show that the structures converge to the same overall architecture within the limits of the resolution. (C) We tested the reliability of our starting model by refining the Rrp5•RNA data against an ellipsoid as a starting model. The final structure is similar in size and shape to Rrp5•RNA aligned against the more accurate RCT-derived starting model.
(TIF)

S1 Table X-ray data collection and refinement statistics. *Values in parentheses are for highest-resolution shell. ^‡^R_merge_ = Σ_h_Σ_I_|I_I_(h)- < I(h) > |/Σ_h_Σ_I_<I(h)>, where I_I_(h) is the I^th^ measurement of reflection h, and < I(h) > is the weighted mean of all measurements of h. *R*_pim_ is the precision-indicating (multiplicity-weighted) *R*_merge_. ^§^R = Σ_h_|F_obs_(h)–F_calc_(h)|/Σ_h_|F_obs_(h)|. R_work_ and R_free_ were calculated using the working and test reflection sets, respectively.
(DOCX)

S2 Table Yeast strains used in this study. (DOCX)

S3 Table Plasmids used in this study. (DOCX)

## Abbreviations

2-D: two dimensional
3-D: three dimensional
3DEM: three-dimensional electron microscopy
ADP: adenosine diphosphate
ATP: adenosine triphosphate
AF: assembly factor
APS: Advanced Photon Source
EJC: exon-junction complex
EM: electron microscopy
FSC: Fourier shell correlation
GSH: glutathione
GST: glutathione S-transferase
ITS: internal transcribed spacer
MBP: maltose binding protein
PDB: Protein Data Bank
RCT: random conical tilt
Rrp5•RNA: Rrp5•H45-3’ITS1
Rrp5•Rok1: Rok1-bound Rrp5
SAD: single wavelength anomalous dispersion
OB fold: oligonucleotide binding fold
snoRNA: small nucleolar RNA
SSRL: Stanford Synchrotron Radiation Lightsource
TAP: tandem affinity purification
TPR: tetratricopeptide
UV: ultraviolet
WT: wild type
