# Supplementary figures and images for: The DEAD-box Protein Rok1 Orchestrates 40S and 60S Ribosome Assembly by Promoting the Release of Rrp5 from Pre-40S Ribosomes to Allow for 60S Maturation
Source: PLoS Biol. 2016 Jun 9;14(6):e1002480. doi: 10.1371/journal.pbio.1002480 (PMC4900678; doi:10.1371/journal.pbio.1002480)

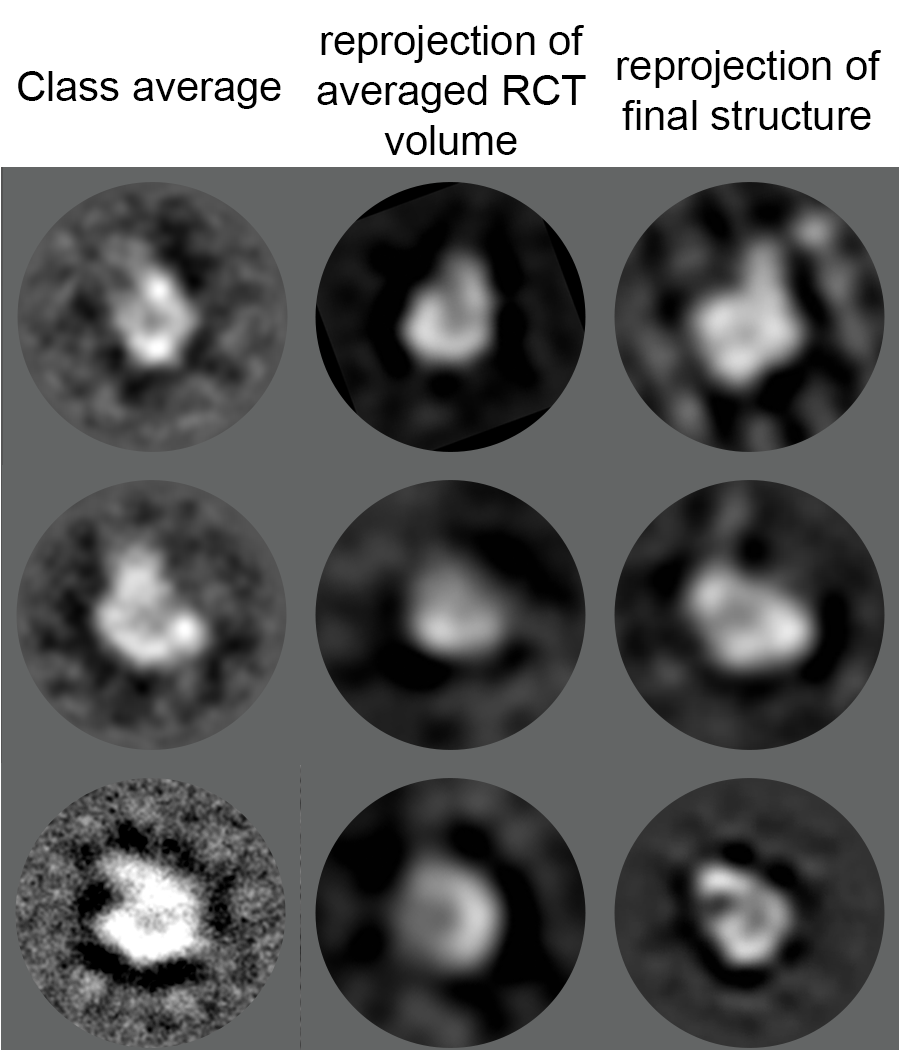

Supplement: S5 Fig — Column 1: Reference-free class averages of negatively stained Rrp5•RNA show a round, fist-like core with a thumb-like projection. Column 2: The reprojection of the averaged RCT volume shows similarity to the averages in column 1. This model was used as the starting model for refining both the Rrp5•RNA and Rrp5•Rok1 structures. Column 3: The corresponding reprojection of the final Rrp5•RNA structure. (TIF) [file pbio.1002480.s006.tif]

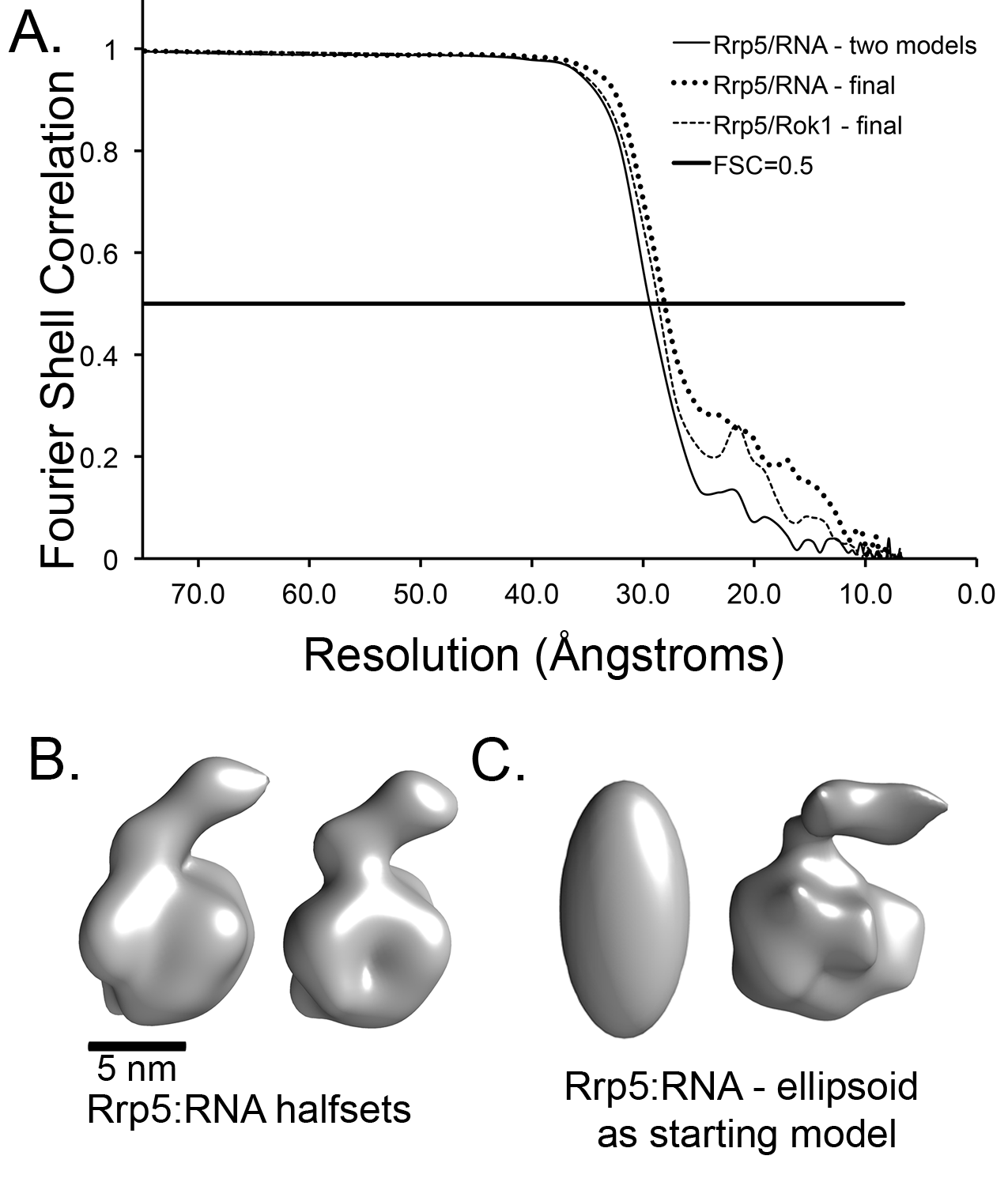

Supplement: S9 Fig — (A) Fourier shell correlation (FSC) analysis of both structures, each refined and reconstructed as two independent half sets. At correlation of 0.5, both are at about 2.8 nm resolution. (B) Two half sets of Rrp5•RNA show that the structures converge to the same overall architecture within the limits of the resolution. (C) We tested the reliability of our starting model by refining the Rrp5•RNA data against an ellipsoid as a starting model. The final structure is similar in size and shape to Rrp5•RNA aligned against the more accurate RCT-derived starting model. (TIF) [file pbio.1002480.s010.tif]
