# Supplementary material for: The DEAD-box Protein Rok1 Orchestrates 40S and 60S Ribosome Assembly by Promoting the Release of Rrp5 from Pre-40S Ribosomes to Allow for 60S Maturation
Source: PLoS Biol. 2016 Jun 9;14(6):e1002480. doi: 10.1371/journal.pbio.1002480 (PMC4900678; doi:10.1371/journal.pbio.1002480)
Supplement: S1 Table — *Values in parentheses are for highest-resolution shell. ‡Rmerge = ΣhΣI|II(h)- < I(h) > |/ΣhΣI<I(h)>, where II(h) is the Ith measurement of reflection h, and < I(h) > is the weighted mean of all measurements of h. Rpim is the precision-indicating (multiplicity-weighted) Rmerge. §R = Σh|Fobs(h)–Fcalc(h)|/Σh|Fobs(h)|. Rwork and Rfree were calculated using the working and test reflection sets, respectively. (DOCX) [file pbio.1002480.s011.docx]

|  | Native | Peak |
| --- | --- | --- |
| Wavelength [Å] | 0.97530Å | 0.97857 Å |
| Resolution [Å] | 57.07-2.7  (2.83-2.7) ^*^ | 56.82-2.9  (3.1-2.9) ^*^ |
| Space group | P 3_1_ 2 1 | P 3_1_ 2 1 |
| Cell parameters:  a,b,c [Å]  α,β,γ [°] | 114.1, 114.1, 67.84  90, 90, 120 | 113.65, 113.65, 67.60  90, 90, 120 |
| R_merge_ ^‡^ [%] | 17.8 (63.8) ^*^ | 12.9 (72.5) ^*^ |
| R_pim_ [%] | 8.6 (31.9) ^*^ | 5.5 (30.8) ^*^ |
| *I* / δ*I* | 7.5 (3.0) ^*^ | 11.6 (2.4) ^*^ |
| *CC_(1/2)_* [%] | 99.3 (84.8) ^*^ | 99.8 (87.6) ^*^ |
| Completeness [%] | 99.9 (100.0) ^*^ | 100 (100) ^*^ |
| Redundancy | 9.9 (9.5) ^*^ | 12.3 (12.5) ^*^ |
| **Refinement** |  |  |
| Resolution [Å] | 43.67-2.70 (2.8-2.7) ^*^ |  |
| No. reflections | 14280 |  |
| *R*_work_ / *R*_free_ ^§^ [%] | 22.11/28.26 |  |
| No. atoms |  |  |
| Protein | 2258 |  |
| Ligand | 6 |  |
| Water | 54 |  |
| *B*-factors [Å^2^] |  |  |
| Protein | 41.359 |  |
| Ligand | 46.488 |  |
| Water | 27.491 |  |
| Ramachandran Plot (%) |  |  |
| Preferred region | 97.8 |  |
| Allowed region | 2.2 |  |
| Outliers | 0.0 |  |
| R.m.s deviations |  |  |
| Bond lengths (Å) | 0.008 |  |
| Bond angles (°) | 1.008 |  |
| PDB code | 5C9S |  |
