# Supplementary material for: The DEAD-box Protein Rok1 Orchestrates 40S and 60S Ribosome Assembly by Promoting the Release of Rrp5 from Pre-40S Ribosomes to Allow for 60S Maturation
Source: PLoS Biol. 2016 Jun 9;14(6):e1002480. doi: 10.1371/journal.pbio.1002480 (PMC4900678; doi:10.1371/journal.pbio.1002480)
Supplement: S2 Table — (DOCX) [file pbio.1002480.s012.docx]

| Strain | Description | Genotype | Reference |
| --- | --- | --- | --- |
| yKK183 | Rrp5TAP | Rrp5TAP::HIS3 | Thermofisher |
| yKK91 | Enp1TAP | Enp5TAP::HIS3 | Thermofisher |
| yKK305 | Rrp5TAP, Gal::Rok1 | Rrp5TAP::HIS3  GalRok1::KanMX6 | This work |
| yKK520 | Enp1TAP, Gal::Rok1 | Enp1TAP::HIS3  GalRok1::KanMX6 | This work |
| yKK644 | Enp1TAP, Gal::Rrp5 | Enp1TAP::HIS3  GalRrp5::KanMX6 | This work |
| yKK248 | Gal::Rrp5TAP | Rrp5TAP::HIS3  GalRrp5TAP::KANMX6 | This work |
