# Supplementary material for: The DEAD-box Protein Rok1 Orchestrates 40S and 60S Ribosome Assembly by Promoting the Release of Rrp5 from Pre-40S Ribosomes to Allow for 60S Maturation
Source: PLoS Biol. 2016 Jun 9;14(6):e1002480. doi: 10.1371/journal.pbio.1002480 (PMC4900678; doi:10.1371/journal.pbio.1002480)
Supplement: S3 Table — (DOCX) [file pbio.1002480.s013.docx]

| Plasmid name | Description |
| --- | --- |
| pKK3298 | pRS416TEF-Rok1 (FL) |
| pKK3310 | pRS416TEF Rok1 (K172A) |
| pKK3299 | pRS416TEF Rok1 (D280A) |
| pKK1074 | pET23b-Rrp5 (FL) |
| pKK3129 | pRS416TEF-FLRrp5 |
| pKK3617 | pRS416TEF-FLRrp5_TPR2  (E1509K/E1510K/E1512K) |
| pKK3620 | pRS416TEF-FLRrp5_TPR7  (K1668E/K1689E) |
| pKK3625 | pRS416TEF-FLRrp5_TPR2+7 |
| pKK3686 | pRS416TEF-Rrp5_N12 |
| pKK3683 | pRS416TEF-Rrp5_N9 |
| pKK3679 | pRS416TEF-Rrp5_C8 |
| pKK3632 | pRS416TEF-Rrp5_C7 |
| pKK1411 | pET23b-Rrp5 (TRP2+7) |
| pKK1421 | pGEX6P3-Rrp5 (C2) |
| pKK1375 | pGEX6P3-Rrp5 (C8) |
| pKK1376 | pGEX6P3-Rrp5 (C7) |
| pKK1375 | pGEX6P3-Rrp5 (C6) |
| pKK1366 | pGEX6P3-Rrp5 (C5) |
| pKK1031 | pSV272-Rok1 (FL) |
| pKK1072 | pET23a-Rrp5 (N9) |
| pKK1345 | pET23a-Rrp5 (N12) |
| pKK1417 | pSV272-Has1 |
